# The Effects of Rearfoot Position on Lower Limb Kinematics during Bilateral Squatting in Asymptomatic Individuals with a Pronated Foot Type

**DOI:** 10.2478/v10078-012-0001-0

**Published:** 2012-04-03

**Authors:** Valerie Power, Amanda M. Clifford

**Affiliations:** 1 Department of Clinical Therapies, Faculty of Education and Health Sciences, University of Limerick, Ireland.

**Keywords:** orthotics, squat, lower limb, kinematics, pronation

## Abstract

Clinicians frequently assess movement performance during a bilateral squat to observe the biomechanical effects of foot orthotic prescription. However, the effects of rearfoot position on bilateral squat kinematics have not been established objectively to date. This study aims to investigate these effects in a population of healthy adults with a pronated foot type.

Ten healthy participants with a pronated foot type bilaterally (defined as a navicular drop >9mm) performed three squats in each of three conditions: barefoot, standing on 10mm shoe pitch platforms and standing on the platforms with foam wedges supporting the rearfoot in subtalar neutral. Kinematic data was recorded using a 3D motion analysis system. Between-conditions changes in peak joint angles attained were analysed.

Peak ankle dorsiflexion (p=0.0005) and hip abduction (p=0.024) were significantly reduced, while peak knee varus (p=0.028) and flexion (p=0.0005) were significantly increased during squatting in the subtalar neutral position compared to barefoot. Peak subtalar pronation decreased by 5.33° (SD 4.52°) when squatting on the platforms compared to barefoot (p=0.006), but no additional significant effects were noted in subtalar neutral.

Significant changes in lower limb kinematics may be observed during bilateral squatting when rearfoot alignment is altered. Shoe pitch alone may significantly reduce peak pronation during squatting in this population, but additional reductions were not observed in the subtalar neutral position. Further research investigating the effects of footwear and the subtalar neutral position in populations with lower limb pathology is required.

## Introduction

Excessive pronation of the foot during exercise has frequently been cited as a risk factor for lower limb injury (Neely, 1998; Buist et al., 2010). The clinical benefits of foot orthotics in the prevention and alleviation of such injuries have been reported in a variety of populations who exhibit excessive pronation and among a range of lower limb pathologies (Nigg et al., 1999). The underlying causes of these effects are not yet fully understood, since foot orthotics have been found to influence a number of variables. In a recent review, Mills et al. (2009) attempted to broadly categorise the physiological bases for the beneficial effects of foot orthotics in terms of kinematic, shock attenuation and neuromotor control paradigms, while acknowledging that a consensus has yet to be reached on the precise roles and interactions of each of these factors.

In terms of kinematic effects, the lack of uniformity in prescription methods used in the current literature renders comparison of findings between studies difficult (Mundermann et al., 2003). Evidence is also lacking in relation to many specific foot orthotics prescription techniques (Ball and Afheldt, 2002b). Thus it is difficult for clinicians to reach definitive conclusions regarding best practice in foot orthotics prescription. Gross et al. (1991) observed the potential detrimental effect on patient care which this may have, reporting that 13.5% of participants in their study experienced increased severity of symptoms or developed new complaints with custom foot orthotic usage due to poor fitting or diagnosis. Therefore, research using well-defined prescription methods is required to enable valid comparison of orthotic effects and ensure the best interests of patients are being met.

**Figure 1:**
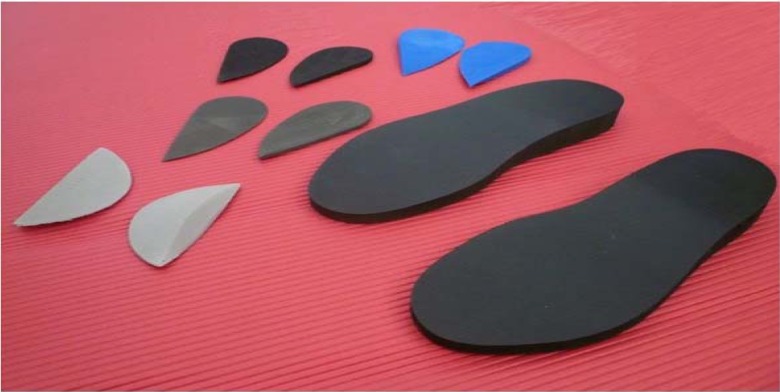
Neutral Zone Prescription Platform (NZPP), consisting of 10mm shoe pitch platforms, foam arch supports and medial rearfoot wedges of varying degrees

Methods of foot posture and orthotics assessment which incorporate analysis of the dynamic, weight-bearing characteristics of the foot are increasingly being favoured by clinicians due to their functional relevance (Ball and Afheldt, 2002a). This has been achieved in this study through the use of the Neutral Zone Prescription Platform (NZPP) (PPL Biomechanics, Cork, Ireland) to assess and alter alignment of the foot. This novel method utilises a combination of solid platforms with a 10mm shoe pitch, foam arch supports and rearfoot wedges to provide individualised correction of rearfoot alignment to subtalar neutral in weight-bearing (Figure 1). Once applied, it is recommended that the effectiveness of the prescription is objectively tested using dynamic functional tests, including a bilateral squat (PPL Biomechanics, 2006). The bilateral squat is used particularly to screen for the occurrence of dynamic knee valgus, the presence of which has been identified as a risk factor for sustaining acute knee injuries during physical activity (Chaudhari and Andriacchi, 2006; Hewett et al., 2005).

The reliability of this method for correcting the rearfoot to a subtalar joint neutral position has been found to be moderate to high (McNamara and Clifford, 2009). However, the resulting changes in alignment and dynamics of the lower limb which these alterations bring about have not been evaluated previously. More specifically, the effects of this correction on bilateral squat kinematics are not understood despite the recommended use of this task as a functional test of the prescription.

Therefore, the aim of this study is to determine what effects, if any, changes in rearfoot alignment have on ankle, subtalar, knee and hip joint kinematics during bilateral squatting in asymptomatic individuals who pronate, since individuals of this foot posture are likely to use foot orthotics and benefit from their usage (Ball and Afheldt, 2002a). It is hypothesised that altering rearfoot alignment will bring about kinematic changes in the lower limb which may be observed during bilateral squatting, and that these changes will be in line with those reported in previous studies of custom foot orthotics.

## Material & Methods

### Participants

Ten participants were randomly selected from a sample of 28 eligible volunteers identified via recruitment e-mail sent to a population of University staff and students. Approval was granted by the University of Limerick Research Ethics Committee and all participants provided written informed consent prior to participation. Exclusion criteria included a history of musculoskeletal or neurological conditions which impair lower limb movement, lower limb injury within the previous six months, or current foot or leg pain. These criteria were selected as they may alter movement patterns of the lower limbs and therefore act as confounding variables in kinematic measurement. A pronated foot posture was the primary inclusion criterion for this study, the presence of which was determined using the navicular drop test (Sell et al., 1994). The navicular drop test measures pronation in terms of the change in height of the navicular bone of the foot between non-weight-bearing and weight-bearing positions, and has been proven to be a valid and reliable non-invasive measure of foot posture (Billis et al., 2007). Normal values of 5–9mm have been established; therefore only participants with a navicular drop of greater than 9mm were included in this study as this was deemed indicative of excessive pronation (Cote et al., 2005). The reliability of the navicular drop test as performed by the investigator in this study was tested prior to commencement of the study.

### Materials & Equipment

The NZPP was used to alter the rearfoot alignment of participants in standing. Participants were required to stand on a set of solid platforms which replicate the 10mm shoe pitch gradient of a standard shoe. Subtalar joint neutral position was assessed by palpating the anteromedial and anterolateral aspects of the talar head with the thumb and index finger while asking the participant to alternately roll their foot into pronation and supination. Subtalar joint neutral was determined when the talar head was felt equally between the thumb and index finger, as described by Sell et al. (1994). Foam arch supports (low, medium or high) were applied as appropriate. The height applied was determined by assessing the conformity of the foam support with the participant’s natural arch profile in a non-weight-bearing subtalar joint neutral position. Rearfoot medial wedges of varying degrees were then applied as appropriate such that the participant could maintain subtalar joint neutral in relaxed stance. The investigator in this study was trained in the use of the NZPP and reliability was tested prior to commencing the study.

Kinematic data was recorded using the CODA mpx64 motion analysis system (Charnwood Dynamics Ltd., Leicestershire, UK), a reliable and valid system for measuring lower limb dynamics (Richards, 1999; Maynard et al., 2003; Monaghan et al., 2007). The CODA mpx64 is a three-dimensional pre-calibrated system which uses optical sensors to capture infra-red light signals emitted by markers placed on specific anatomical landmarks of participants. The markers were applied by one investigator in line with manufacturer’s guidelines. Kinematic data was recorded at a 200Hz sampling rate for 5 seconds for each squatting trial performed.

### Testing Procedure

Each participant presented for a single testing session during which screening and all trials were completed. Once screened for eligibility, the required LED markers were applied and participants stood on soft thin rubber mats to aid marker visibility. Participants were asked to adopt their normal stance, the width of which was marked to aid placement of the NZPPs later in testing. Participants were then asked to perform a bilateral squat to their maximal range while keeping their heels on the floor. A brief demonstration was provided by the investigator. Participants crossed their arms across their chests during squatting to prevent obstructing marker visibility. No additional instructions regarding squatting technique were provided to ensure that participants adopted their individual natural movement patterns.

**Figure 2:**
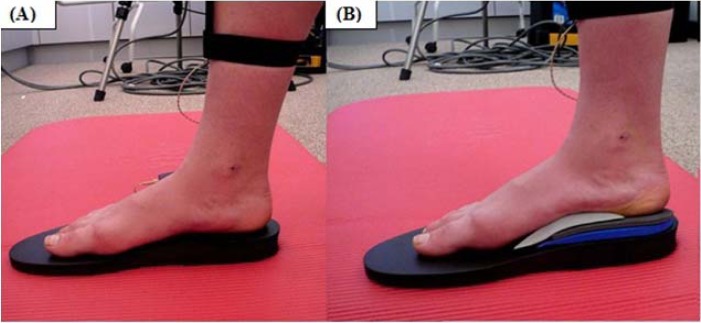
(A) PLT condition indication foot position standing on the NZPP. (B) COR condition, with foam arch support and medial rearfoot wedges in place

Participants undertook a minimum of three practice squats prior to recording in each condition in order to become familiar with the task and equipment. Kinematic data was recorded for three bilateral squats under each condition: barefoot (BFT), standing on the 10mm shoe pitch platform only (PLT), and corrected stance on the platform in the subtalar neutral position with arch supports and medial rearfoot wedges in place (COR) (Figure 2). Trials were conducted in this order for all participants.

### Data Analysis

**Figure 3:**
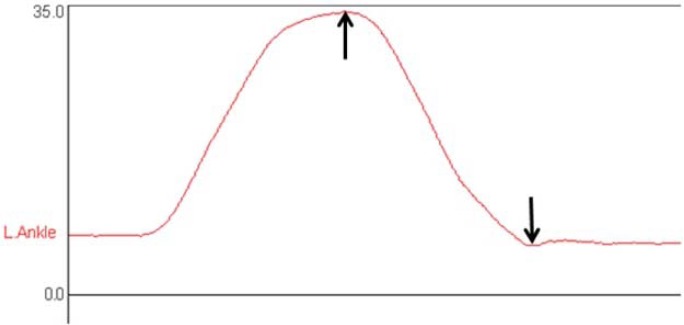
Sample graph obtained using the Codamotion Analysis Software indicating the peak left ankle dorsiflexion (uppermost arrow) and plantarflexion (lowermost arrow) values obtained during squatting in the PLT condition

Kinematic data for the peak subtalar, ankle, knee and hip joint angles achieved during squatting were obtained using the Codamotion Analysis software package. Peak values that occurred throughout the squat were obtained as illustrated in Figure 3. Data from each lower limb of each participant was analysed independently (n=20), since participants demonstrated side-to-side differences in the amount of pronation and correction applied, and also in kinematics during squatting.

A statistical analysis was performed using SPSS Version 16.0 for Windows. For data sets which were normally-distributed, parametric testing to identify differences in peak joint angles across all conditions was performed using a oneway repeated measures analysis of variance (ANOVA). Post hoc paired t-tests with a Bonferroni correction were then performed to compare differences between individual pairs of conditions. For data sets which were not normally-distributed, non-parametric testing was performed using Friedman’s tests to evaluate changes across all conditions. Post hoc Wilcoxon signed ranks tests were used to detect significant differences between specific pairs of conditions.

## Results

### Preliminary Reliability Testing

Reliability of the navicular drop test, as performed by the rater in this study, was assessed prior to testing. Good intra-rater reliability (ICC = 0.798; 95% CI 0.377 to 0.946) and inter-rater reliability (ICC = 0.743; 95% CI 0.254 to 0.929) were found, with mean differences (standard deviation) of 1.7mm (2.1mm) and 2.2mm (2.3mm) respectively.

A preliminary investigation of the reliability of applying rearfoot correction using the NZPP was also conducted prior to testing. Excellent intra-rater (ICC = 0.986; 95% CI = 0.933 to 0.997) and inter-rater reliability (ICC = 0.853; 95% CI = 0.431 to 0.969) were found, with mean differences of 0.25° (0.886°) and 0° (2.726°) respectively.

#### Participant Characteristics

A summary of participants’ characteristics is presented in Table 1.

### Ankle/Subtalar Joint Kinematics

A summary of the changes in peak ankle/subtalar joint angles attained during squatting is provided in Table 2. ANOVA results indicated statistically significant differences across all three conditions in peak plantarflexion (*p* < 0.0005), dorsiflexion (*p* < 0.0005) and pronation (*p* = 0.009). A non-significant increase in peak supination was noted across all conditions, with pairwise comparisons detecting significance between BFT and PLT.

### Knee Kinematics

A statistically significant increase in peak knee flexion was noted across all conditions (Table 2). Changes in peak knee varus were significant between BFT and PLT only. No significant changes in peak knee extension or valgus angles were noted.

### Hip Kinematics

A significant difference in peak hip abduction was observed across the three conditions (Table 2), with pairwise comparisons indicating statistically significant decreases in peak hip abduction between BFT and COR conditions, and between PLT and COR.

## Discussion

Statistically significant alterations in kinematics at the ankle, subtalar, knee and hip joints were observed during bilateral squatting under varying conditions, although changes were predominantly seen at the ankle/subtalar joint complex.

### Ankle/Subtalar Kinematics

A reduction in the magnitude of stance-phase pronation has been reported with foot orthotic use in numerous studies examining gait (McCulloch et al., 1993; Eng and Pierrynowski, 1994; MacLean et al., 2006).

Although a different weight-bearing task was analysed in this study, peak pronation was similarly seen to decrease in the PLT and COR conditions compared to BFT. These decreases in peak pronation are seen to be broadly matched by increases in peak supination, a finding which mirrors those of McCulloch et al. (1993) and Nawoczenski et al. (1995). These studies noted that total frontal plane range of motion remained unchanged with orthotic use, indicating that motion is not limited by orthotic usage but rather repositioned in range towards supination.

Although the mean reduction in peak pronation observed in this study marginally failed to reach statistical significance between BFT and COR (*p* = 0.054), the observed value of 4.64° was in line with the 2–4° reductions in peak pronation noted in previous studies of foot orthotics (Mills et al., 2009). Clinical benefits have been attributed to such reductions in peak pronation in a variety of lower limb pathologies among athletes and other patient populations (Nigg, 2001), suggesting that, despite failing to reach statistical significance, the effects observed in this study may be of clinical significance. However, the large standard deviation of the mean (8.01°) also suggests that effects varied greatly between participants.

Pairwise comparisons revealed a non-significant increase in peak pronation and corresponding decrease in peak supination between PLT and COR. Williams et al. (2003) noted similar findings, with 46% of participants in their study exhibiting increased peak pronation when wearing orthotics compared to no orthotics. The authors postulated that this occurrence may be due to participants who habitually wear orthotics experiencing feelings of instability when performing activities without orthotic support. They suggested that such individuals may compensate by actively supinating in the absence of orthotics, thereby causing the orthotics condition to appear relatively pronated compared to the no orthotics condition. A similar occurrence may have contributed to our findings, since participants were included regardless of orthotics usage. The compliant nature of the mat on which BFT squats were performed may have offered greater support to the foot than the rigid platform, rendering participants more subjectively unstable – and thus more likely to actively supinate – in the PLT condition rather than BFT. The large reduction in peak pronation between BFT and PLT of 5.33° may support this concept, as it may be explained in part by this proposed compensatory supination in the PLT condition.

Highly significant changes in sagittal plane kinematics were noted, with peak ankle plantarflexion increasing and peak dorsiflexion decreasing across all conditions. Such changes are to be expected considering the progressive heel lifts provided by the platform and rearfoot wedges (Figure 2). This reciprocal relationship is similar to that observed in the coronal plane and indicated a shift in movement pattern towards plantarflexion.

The kinematic changes observed at the subtalar and ankle joints must not be considered in isolation. Plantarflexion of the ankle and supination of the subtalar joint are biomechanically linked, thus plantarflexion may be a source of the anti-pronatory effect of heel lifts (Hirth, 2007). Muscular structures may contribute to the kinematic effects of heel lifts, since tightness of lateral ankle musculature – lateral gastrocnemius, soleus and peroneals – can promote tibial abduction and external rotation, precipitating foot pronation and knee valgus (Hirth, 2007). Conversely, weak medial gastrocnemius, tibialis anterior and tibialis posterior may decrease the ability to control foot pronation and knee valgus. Hirth (2007) posited that a heel lift can decrease tension within lateral structures, thereby restoring normal length-tension relationships between medial and lateral ankle musculature and ultimately optimising alignment and dynamic control during squatting. Bell et al.’s (2008) findings support these hypotheses. They found that participants who experienced dynamic knee valgus during bilateral squatting presented with decreased plantarflexor strength and also plantarflexor tightness, exhibited as an ankle dorsiflexion range of motion deficit of approximately 20% compared to those with normal squat patterns. These findings, together with the findings of this study, suggest a potential role for foot orthotics usage among athletes who frequently perform squatting activities in training but who struggle to maintain optimal lower limb alignment and dynamic control during these activities.

### Knee Kinematics

Of greatest significance at the knee joint were the increases in peak flexion seen across all conditions. Since increasing ankle plantarflexion reduces Achilles tendon tension, a greater squat depth – manifested in our results as increased knee flexion – may be attained by participants who were previously limited by plantarflexor muscle length (Hirth, 2007).

Peak knee varus increased significantly between BFT and COR (*p* = 0.028). This 1.13° increase is comparable to the 1.5–2° increases in peak knee varus observed by Williams et al. (2003) with increasing amounts of medial rearfoot posting. These changes suggest that orthotic interventions which reduce rearfoot eversion may have a clinical role to play in reducing stress on medial knee structures and compression of the lateral compartment of the knee joint, and may aid in preventing or alleviating potential resultant knee injuries during squatting exercise.

Despite observing a mean difference in peak knee varus, no significant effect on peak knee valgus was seen. This finding does not definitively indicate that dynamic knee valgus was unchanged by altering rearfoot position, since analysis of the timing of peak varus and valgus during squatting would be required to give a clear representation of any potential effects. It must also be noted that variability in the effects on frontal plane kinematics was high, with standard deviations of the mean differences relatively large. Such high variability is echoed throughout the current literature (Eng and Pierrynowski, 1994; McCulloch et al., 1993; MacLean et al., 2006). Thus, it is not possible to draw definitive conclusions regarding the subtle changes in peak frontal plane kinematics found in this study. Further studies examining a range of dynamic activities are required to reach a consensus on the effects of rearfoot position on frontal plane knee motion or to identify specific subgroups that vary in response to changes in rearfoot position.

### Hip Kinematics

Our results indicated few significant kinematic alterations at the hip joint across conditions. A statistically significant decrease in peak hip abduction was identified between PLT and COR, and BFT and COR conditions. The clinical significance of these changes is highly questionable however, since they equated to an absolute change of approximately 1°, and are accompanied by relatively large standard deviations of 1.85° and 1.49° respectively.

It is also worth noting the lack of significant change in peak hip adduction observed. According to Hirth (2007), the changes in peak pronation and supination observed at the foot may theoretically lead to proximal effects in the form of decreased peak knee valgus and peak hip adduction. Neither was observed in this study. The most notable feature of our results in relation to peak hip adduction was the high variability of effects between participants.

#### Implications

It is apparent from our results that alterations in rearfoot alignment may bring about significant changes in lower limb kinematics during squatting. Certain biomechanical effects of altering rearfoot alignment, which have been linked to clinical benefits in patients who display excessive pronation, have been reproduced in this study. The decreases in peak pronation observed suggest a role for the usage of orthotics which alter rearfoot alignment among athletes with excessively pronated foot posture, since these changes have been linked to prevention and relief of many lower limb pathologies (Nigg, 2001). The increases in peak knee varus observed may reflect a reduction in dynamic knee valgus, and thus may lead to decreased risk of knee injury (Chaudhari and Andriacchi, 2006; Hewett et al., 2005). This observation may be particularly relevant to athletes who frequently engage in squatting during training or competitive activities. The changes in lower limb kinematics noted in this study – though small – may be clinically relevant to those undertaking a high volume of repetitive motions e.g. during gait or specific training exercises, due to the potential cumulative effects (Nawoczenski et al., 1995).

The significance of the effects observed in the PLT condition suggest that the heel lift aspect of the procedure to alter rearfoot alignment contributed most greatly to the overall effects observed in this study. Shoe pitch or the degree of heel lift applied may therefore be important for athletes to consider when selecting footwear or seeking foot orthotics for use during exercise. Further research investigating the relative contributions of shoe pitch and subtalar neutral positioning to kinematics of other tasks is required to establish if our results are generalizable to the performance of other functional activities.

Our results confirm the utility of the bilateral squat to clinicians as a functional test when prescribing foot orthotics to patients or athletes using the NZPP, since kinematic changes throughout the lower limb – such as reduced pronation of the foot, increased squat depth and increased knee varus – were observed during this activity. These potentially beneficial kinematic changes are similar to those found among orthotics fabricated using other prescription methods (Mills et al., 2009), suggesting that the NZPP may constitute clinically valuable means of assessing and prescribing custom foot orthotics.

### Limitations

Certain limitations existed in the design of this study. Firstly, it has been proposed that the effects of foot orthotics on the temporal parameters of movement may be significant in producing clinical benefits (McCulloch et al., 1993; MacLean et al., 2006). In this study the peak joint angle achieved was measured for each movement, but the timing of this peak could not be analysed accurately within the protocol employed. Thus, potentially significant effects on timing may have gone undetected.

Rotations about the longitudinal axis were also not analysed in this study. However, Nawoczenski et al.’s (1995) findings suggest that a specific relationship exists between lower limb movements that measures of tibial rotation reflect subtalar joint pronation and supination in weight-bearing, and vice-versa. Because of this movement coupling, it was not necessary to analyse tibial rotations independently, since inferences regarding the effects on tibial rotation may be made based on the effects noted at the subtalar joint.

Participants were drawn from a healthy population; therefore the effects noted may differ in injured populations where pain may also contribute to alterations in lower limb kinematics. Current studies of orthotic effects in injured populations frequently lack specificity in categorising the types of injuries included. Therefore, further research is required to evaluate effects in populations with specific lower limb injuries.

The use of the NZPP to correct rearfoot position limited the potential tasks for kinematic analysis since participants had to remain standing on the NZPP. While the effects noted in this task are directly applicable to methods of clinical assessment and prescription of foot orthotics using the NZPP, the authors acknowledge the limitations regarding extrapolating these results to other dynamic functional tasks. Future studies which analyse the effects on functional tasks of custom foot orthotics prescribed using this prescription method are required to gain full understanding of the potential clinical influences of such devices.

## Conclusions

Our results confirm our hypothesis that altering rearfoot alignment would bring about kinematic changes throughout the lower limb during squatting. The kinematic changes observed may reduce excessive pronation and dynamic knee valgus. Based on current evidence, these changes may help in preventing and/or managing lower limb injury in athletes or patients who pronate excessively, particularly those who frequently engage in squatting activities during training and competition. It appears that shoe pitch alone may bring about significant reductions in pronation without additional rearfoot posting to obtain a subtalar neutral position, but further research is needed to confirm if this finding can be generalised to the wider population, to injured populations and to other activities aside from squatting.

Alterations in lower limb kinematics were observed during a bilateral squat, thus this task may be of use in clinical practice as a functional test of the prescription of foot orthotics. The kinematic changes observed when rearfoot correction was applied using the NZPP were similar to those noted in studies of custom foot orthotics prescribed using other methods, which suggests that the NZPP may constitute valuable means of assessing and prescribing custom foot orthotics. Future studies investigating the effects of custom foot orthotics prescribed using this method on dynamic functional activities e.g. gait, are required.

## Figures and Tables

**Table 1:** Characteristics of participants.

Gender	4 male, 6 female
Age; mean (SD)	21 years (1.2 years)
Navicular drop; mean (SD)	10.9mm (2.06mm)
Rearfoot correction applied; mean (SD)	12.7° (3.87°)

SD = standard deviation.

**Table 2:** Mean differences in peak joint angles attained at the ankle, subtalar, knee and hip joints between conditions.

	BFT/PLT Mean (SD)	*p*	PLT/COR Mean (SD)	*p*	BFT/COR Mean (SD)	*p*
*Ankle*						
PF	6.53°(3.37°)	0.0005^*^	3.26°(2.93°)	0.0005^*^	10.06°(5.29°)	0.0005^*^
DF	−4.00°(2.72°)	0.0005*	−2.94°(3.32°)	0.003^*^	−6.93°(4.05°)	0.0005^*^
*Subtalar*						
Pron	−5.33°(4.52°)	0.006^*^	0.69°(4.32°)	1	−4.64° (8.01°)	0.054
Sup	4.89°(6.41°)	0.004^*^	−1.31°(5.71°)	0.191	3.58° (10.15°)	0.332
*Knee*						
Flex	4.53°(6.57°)	0.014^*^	5.48°(7.26°)	0.004^*^	10.01° (12.45°)	0.005^*^
Ext	0.35°(1.69°)	1	0.20°(1.56°)	1	0.56° (1.90°)	0.62
Varus	0.80°(1.76°)	0.021^*^	0.34°(1.96°)	0.059	1.13° (2.56°)	0.028^*^
Valgus	0.18°(4.12°)	1	−0.13°(1.65°)	1	0.05° (4.33°)	1
*Hip*						
Flex	1.37° (5.31°)	0.681	0.85° (4.37°)	0.681	2.22° (8.62°)	0.455
Ext	0.02° (2.08°)	1	−0.12° (2.43°)	1	−0.10° (2.79°)	1
Abd	0.18° (1.80°)	1	−1.17° (1.85°)	0.032^*^	−0.99° (1.49°)	0.024^*^
Add	−0.24° (2.32°)	1	−0.28° (8.36°)	1	−0.52° (8.66°)	1

BFT = barefoot; PLT = platform only; COR = platform plus rearfoot correction; SD = standard deviation; p = significance value; PF = plantarflexion; DF = dorsiflexion; Pron = pronation; Sup = supination; Flex = flexion; Ext = extension; Abd = abduction; Add = adduction.

^*^= P<0.05
